# A framework for assessing the risk of resistance for anti-malarials in development

**DOI:** 10.1186/1475-2875-11-292

**Published:** 2012-08-22

**Authors:** Xavier C Ding, David Ubben, Timothy NC Wells

**Affiliations:** 1 Medicines for Malaria Venture, 20 rte de Pré Bois, Geneva, CH 1215, Switzerland

**Keywords:** Resistance, *P. falciparum*, Drug development, Risk assessment

## Abstract

Resistance is a constant challenge for anti-infective drug development. Since they kill sensitive organisms, anti-infective agents are bound to exert an evolutionary pressure toward the emergence and spread of resistance mechanisms, if such resistance can arise by stochastic mutation events. New classes of medicines under development must be designed or selected to stay ahead in this vicious circle of resistance control. This involves both circumventing existing resistance mechanisms and selecting molecules which are resilient against the development and spread of resistance. Cell-based screening methods have led to a renaissance of new classes of anti-malarial medicines, offering us the potential to select and modify molecules based on their resistance potential. To that end, a standardized *in vitro* methodology to assess quantitatively these characteristics in *Plasmodium falciparum* during the early phases of the drug development process has been developed and is presented here. It allows the identification of anti-malarial compounds with overt resistance risks and the prioritization of the most robust ones. The integration of this strategy in later stages of development, registration, and deployment is also discussed.

## Background

Resistance is a phenomenon common to all anti-infective agents that can be defined as a genetically encoded reduction in efficacy of a drug. Anti-malarial medicines are amongst the most commonly used drugs worldwide, and historically the supervision of their administration has been relatively unsupervised. This combination of factors has led to the successive demise of first line treatments such as chloroquine, proguanil, pyrimethamine, sulphadoxine-pyrimethamine and mefloquine, which are unable to produce a 90% clinical response in many areas where they have been deployed intensively [1,2]. Some medicines are clearly more prone to resistance selection than others: resistant strains to chloroquine took decades to emerge, but those to the electron transport inhibitor atovaquone were identified almost in parallel with its first clinical use [3]. This difference has a clear molecular basis: chloroquine resistance requires several mutations in the transporter *pfcrt* (chloroquine resistance transporter), whilst atovaquone resistance requires a single point mutation in the mitochondrially encoded cytochrome bc1 *pfcytb* (cytochrome b) [4]. Altogether, the nature of antimalarial compounds, of their targets, and of the interactions between them ultimately determines the genetic ability of *Plasmodium* parasites to acquire resistance mechanisms. Whether these mechanisms will emerge and spread in the wild further depends on several factors operating at the host and population levels (Table 1). 

**Table 1:** Resistance associated factors

**Factor**	**Level of action**	**Variables**
Drug mode-of-action	Parasite	Target nature (cellular process, protein, other).
		Target gene localization (nuclear or mitochondrial genome).
		Drug subcellular localization (vacuole, organelle, cytoplasm).
Resistance mode-of-action	Parasite	Target mutation rate.
		Nature of mutations required for resistance (single nt, in/del, copy number).
		Number of mutations required for resistance (causal and compensating).
Fitness	Human host	Growth rate of resistant parasite (within host competition)
		Effect of drug on gametocytogenesis and gametocyte viability.
		Effect of resistance mutations on gametocytogenesis and gametocyte viability.
Drug pharmacokinetic	Human host	Clinical parasite reduction ratio
		Drug half-life
		Drug dosage
Drug deployment	Human population	Drug pressure
		Drug combination
		Parasite transmission intensity
		Human population immunity

Fortunately, development of resistance to artemisinin, on which are based current first-line therapies for uncomplicated and severe *P. falciparum* malaria, has been relatively slow. This is partly due to recommendations from the World Health Organization (WHO) that only fixed-dose combinations of artemisinin derivatives with other anti-malarials should be used. It is also because of the relative difficulty of generating artemisinin resistance, which has been reassuringly problematic, even in the laboratory [5]. The first signs of a reduction of the anti-parasitic activity of artemisinins are emerging, with a decrease in parasite clearance time being seen in Cambodia and Thailand, and the first hints of a possible molecular mechanism have been suggested [6-8]. Even if the spread of resistance can be controlled, there will be a need for new classes of anti-malarial drugs. This is a race against time, given that the time taken to develop new medicines often exceeds a decade. Developing new medicines that will withstand resistance is a combination of reducing the possibility that resistant parasites will arise and preventing their transmission. First, finding new molecular classes with a low probability that resistance would arise should be a consequence of more holistic cellular based screening, which has the advantage of potentially selecting for polypharmacology; in other words molecules that hit more than one target. Fitness cost is a key factor in this equation as well: if it is not possible to produce a resistant phenotype with a low fitness cost, then these strains are likely to be replaced by sensitive strains when the drug pressure is relaxed. Second, understanding the role of new inhibitors on transmission is also critical at an early stage, since resistant parasites which are inefficiently transmitted are a far lower threat to global public health concern. Anti-folate resistance had a marked differential effect on transmission which may have accelerated its spread [9]: clearly a molecular class with the opposite trend would be preferable. Third, the spread of resistance can be reduced by combining multiple agents with distinct modes of-action; since any organism resistant to one component of the combination should be eliminated by the other. This raises the barrier for resistance, since the parasite would have to acquire both resistances simultaneously, which is far less likely, assuming the mutation events are stochastic [10]. The combination of artemether-lumefantrine has been protected against the emergence of lumefantrine resistance despite being used to treat over 500 million patients, partly because lumefantrine has never been used as a monotherapy. Fixed dose combinations prevent sub-optimal monotherapy treatment, and will restrict the spread of resistance [10,11], provided the optimal dose ratio can be deployed. Multiple first-line therapies would also help, but the key is to have medicines which are as different as possible and current strategies would only allow use of artemisinin combination therapies [12,13]. Putting together these three factors in models to simulate drug resistance emergence in the wild is an important task, but is still at an early stage [14-16].

The lifespan of the next generation of anti-malarials in the face of resistance is a critical question in the debate over malaria elimination and eradication. If through judicious selection of the right molecules and good practice, the time interval from introduction to clinically significant resistance for the next generation of therapy can be put at the level of artemisinin, or even better, it is possible that the disease will be eliminated before clinical resistance occurs. This increases the need to have an early assessment of the risk of developing resistance associated with each compound and each combination. This would not be the single decision criterion, but would be part of the overall framework of deciding which compounds to prioritize. The Medicines for Malaria Venture (MMV) is a product development partnership with the mission to discover, develop, and facilitate delivery of new, effective and affordable anti-malarial medicines. Part of the process of developing new medicines is to have a coherent view on the required properties of new medicines, a process often referred to as developing the “target product profile” or TPP. Since one key aspect of this product profile has to be minimal susceptibility to resistance, it is important to have a standardized framework to quantitatively assess the risk of resistance associated with preclinical anti-malarial compounds. Such a framework, based on *in vitro P. falciparum* experiments, has been developed and is presented. The experimental strategy is discussed together with the benefits and limitations of such a standard approach and its implications for clinical development.

## Resistance assessment strategy

**Figure 1:**
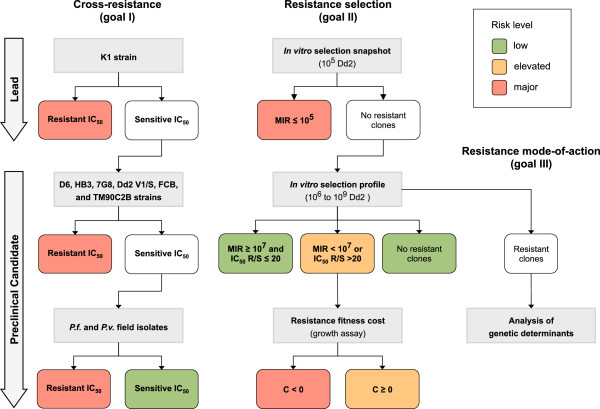
**Resistance risk assessment workflow.** The resistance risk assessment workflow encompasses three goals: cross-resistance determination (goal I), *de novo* resistance selection frequency determination (goal II), and resistance mode-of-action determination (goal III). These can be achieved trough a straightforward set of quantitative experiments applied to compounds at the lead and preclinical developmental stages. A resistant IC_50_ corresponds to a 20-fold increase as compared to a fully sensitive strain (NF54 or HB3 in the case of sulphonamides). C is the theoretical cost of fitness associated with resistance (see main text). C<0 indicates that resistance provides a fitness advantage, which is a major risk factor. Ultimately, the overall risk level can be classified as low, elevated, or major and allows to prioritize the development of robust compounds and to establish risk mitigation strategies for the others.

The MMV assessment strategy has been designed to achieve three goals: (i) identify potential cross-resistance to other anti-malarial compounds and naturally occurring phenotypic differences, (ii) quantify the risk, or frequency, of *de novo* resistance selection *in vitro* and the associated fitness cost, (iii) characterize the molecular mode-of-action of resistance, including an understanding of its genetic basis. This work goes on in parallel with the optimization and selection of candidate molecules. Clearly not all work needs to be done on all molecules in the discovery pipeline, since the sad truth is that most chemical series do not result in a molecule which can be tested in humans. The first aspect should be analysed when a new chemical series is identified, since this is something that may be optimized by a medicinal chemistry approach. The second aspect is probably series dependent, and requires more intensive measurement, and should wait until a project is at the lead optimization stage. The final steps should be carried out in parallel to the clinical candidate selection, since they are relatively labour intensive. Two test cascades have been defined to achieve the first two goals systematically, the results of which can be exploited to the third one (Figure 1).

## Goal I: cross-resistance

**Figure 2:**
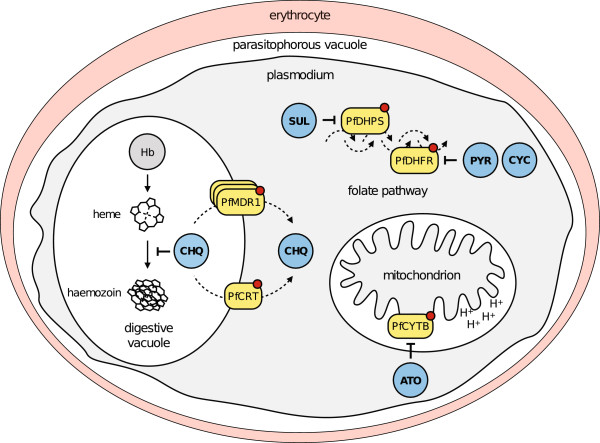
**Known genetic determinants of naturally occurring resistance mechanisms.** Mutations (red dot) of the dihydrofolate reductase (PfDHFR) enzyme prevent its inhibition by the antifolate drugs pyrimethamine (PYR) and cycloguanil (CYC). Similarly, sulphadoxine (SDX) resistance is mediated by mutations of its target dihydropteroate synthetase (PfDHPS). Atovaquone (ATO) binds to the cytochrome *bc*_1_ complex (PfCYTB), mutations of which have been shown to induce high level of ATO resistance. Chloroquine (CHQ) is believed to prevent haeme detoxification within the digestive vacuole. Mutations of the CHQ resistance transporter (PfCRT) as well as of the multidrug resistance protein-1 (PfMDR1), including copy number variations, have been shown to compromise CHQ action by preventing its accumulation within the digestive vacuole. Mutations of these two transporters have also been implicated with mefloquine resistance, although definite marker has not been established for this drug.

One of the hallmarks of genetically determined drug-resistance is a shift in the 50% inhibitory concentration (IC_50_) compared to sensitive strains. The first step is establishing that a new compound is fully active on known multi-drug resistant (MDR) *P. falciparum* strains by measuring its IC_50_, which is a more robust and reproducible measure than IC_90_ or IC_99_. Known anti-malarial resistance is principally determined by only a handful of genes (Figure 2). These include specific molecular targets, such as those for antifolates *pfdhps* (dihydropteroate synthase) and *pfdhfr* (dihydrofolate reductase) and electron transport inhibitors *pfcytb* (cytochrome bc1). In addition there are transporters such as *pfcrt* (chloroquine) and *pfmdr1* (multidrug resistance 1).

A collection of resistant strains has been selected to cover at least all the naturally occurring and genetically defined known resistance mechanisms (Table 2). In this context, we will define resistance as IC_50_ value 20-fold higher than that of the reference IC_50_ value from a pan-sensitive *P. falciparum* strain, typically NF54. This threshold gives room for intrinsic IC_50_ variations in the growth rate or other biological or experimental parameters not directly related to resistance mechanisms [17]. The strains selected cover the majority of known genetic variations. The chloroquine transporter *pfcrt* mutation K76T is associated with additional mutations (CVIET and SVMNT alleles which are found in the FCB and 7G8 strains). The V1/S strain has mutations in *pfdhfr* leading to four amino acid substitutions showing high levels of resistance to cycloguanil and pyrimethamine [18]. HB3 is resistant to pyrimethamine but not cycloguanil, while FCB shows the contrary pattern [18]. Dd2 and FCB show increased copy numbers of *pfmdr1*, and mutations at several codons. These changes influence sensitivity to multiple drugs, including mefloquine and artemisinin derivatives [5,19]. NF54 and 7G8 have a 20-fold shift in IC_50_ to sulphadoxine, as a result of mutations in *pfdhps* whilst Dd2 has a high level resistance, when tested in a low para-aminobenzoic acid (pABA) medium [20,21]. TM90C2B is resistant to atovaquone, due to a mutation in *pfcytb*[22]. Finally the set also includes the D6 strain, which shows, in some studies, a low level of natural resistance to mefloquine [23]. The K1 strain recapitulates most of the natural resistance mechanisms described above and therefore is a good first filter that can be performed early in the screening process. Clearly over the next few years it will be important to add new resistance mechanisms. For example, resistance to the new spiroindolone NITD609 (in Phase IIa) is linked to *pfatp4* as a potential resistance mechanism [24], demonstrating that new classes of compounds can bring new resistance mechanisms. 

**Table 2:** Panel of multidrug resistant strains including specifc amino acid changes

	**drug resistance**^**a**^						***pfcrt***^**b**^					***pfmdr1***^**c**^						***pfdhfr***^**d**^					***pfdhps***^**e**^				***pfcytb***			
**Strain**	**CHQ**	**SUL**	**PYR**	**CYC**	**MEF**	**ATO**	**72**	**73**	**74**	**75**	**76**	**86**	**184**	**1034**	**1042**	**1246**	**copy**	**16**	**51**	**59**	**108**	**164**	**436**	**437**	**581**	**613**	**268**	**Origin**	**MR4 number**	**Ref.**
NF54	S	S	S	S	S	S	C	V	M	N	K	N	Y	S	N	D	1	A	N	C	S	I	S	**G**	A	A	Y	Imported	MRA-100	[25]
D6	S	S	S	S	**R**	S	C	V	M	N	K	N	Y	S	N	D	1	A	N	C	S	I	**A**	A	A	A		Cloned from Sierra Leone I/CDC	MRA-285	[26]
HB3	S	S	**R**	S	S	S	C	V	M	N	K	N	Y	S	**D**	D	1	A	N	C	**N**	I	S	A	A	A		Cloned from Honduras I/CDC	MRA-155	[27]
7 G8	**R**	S	**R**	**R**			**S**	V	M	N	**T**	N	**F**	**C**	**D**	**Y**	1	A	**I**	C	**N**	I	S	**G**	A	A		Cloned from IMTM 22 (Brazil)	MRA-152	[28]
Dd2	**R**	**R**	**R**	**R**	**R**		C	V	**I**	**E**	**T**	**Y**	Y	S	N	D	**4**	A	**I**	**R**	**N**	I	**F**	**G**	A	**S**		Cloned from W2-Mef (Indochina III/CDC)	MRA-150	[29]
V1/S	**R**	**R**	**R**	**R**			C	V	**I**	**E**	**T**	**Y**	Y	S	N	D		A	**I**	**R**	**N**	**L**	**F**	**G**	A	**T**		Cloned from V1 (Vietnam)	MRA-176	[30]
K1	**R**	**R**	**R**	**R**			C	V	**I**	**E**	**T**	**Y**	Y	S	N	D	1	A	N	**R**	**N**	I	S	**G**	**G**	A		Thailand	MRA-159	[31]
FCB	**R**		S	**R**			C	V	**I**	**E**	**T**	**Y**	Y	S	N	D	**2**	**V**	N	C	**T**	I						Columbia	MRA-309	[32]
TM90C2B	**R**		**R**		**R**	**R**	C	V	**I**	**E**	**T**	N	**F**	S	N	D											S	Thailand	N/A	[3]

^a^S: sensitive, R: resistant, as reported in the literature.

^b^Allelic information based on references [33-35].

^c^Allelic information based on references [33,35-37].

^d^Allelic information based on references [18,35,38].

^e^Allelic information based on references [20,39-41].

Since these MDR strains were adapted to culture and cloned many years ago, and have been passaged since that time, they may not represent the wild-type field *Plasmodium* parasites. Moreover, only *P. falciparum* can be stably cultured in the laboratory. It is therefore essential to test compounds against contemporary field isolates of all the human-infecting species, including *Plasmodium vivax, Plasmodium ovale* and *Plasmodium malariae*. This gives good background information on variability, and allows confirmation that the compound is active on all species. The analysis of at least 10 *P. falciparum* and 10 *P. vivax* isolates appears a minimal requirement, if a 20-fold change in IC50 is used as a cut-off [42].

## Goal II: resistance selection frequency

Resistant parasites can be selected by applying drug pressure *in vitro* and *in vivo*[22]. These approaches have successfully identified genes, and sometimes codons involved in naturally occurring resistance [22]. The single nucleotide changes behind these mutations are estimated to occur at a rate of approximately 10^-9^ per nucleotide site per replication, similar to other eukaryotes [43-45]. Changes in sensitivity are also caused by gene copy number variations, which occur as often as 10^-4^ per nucleotide site per replication [45,46].

**Figure 3:**
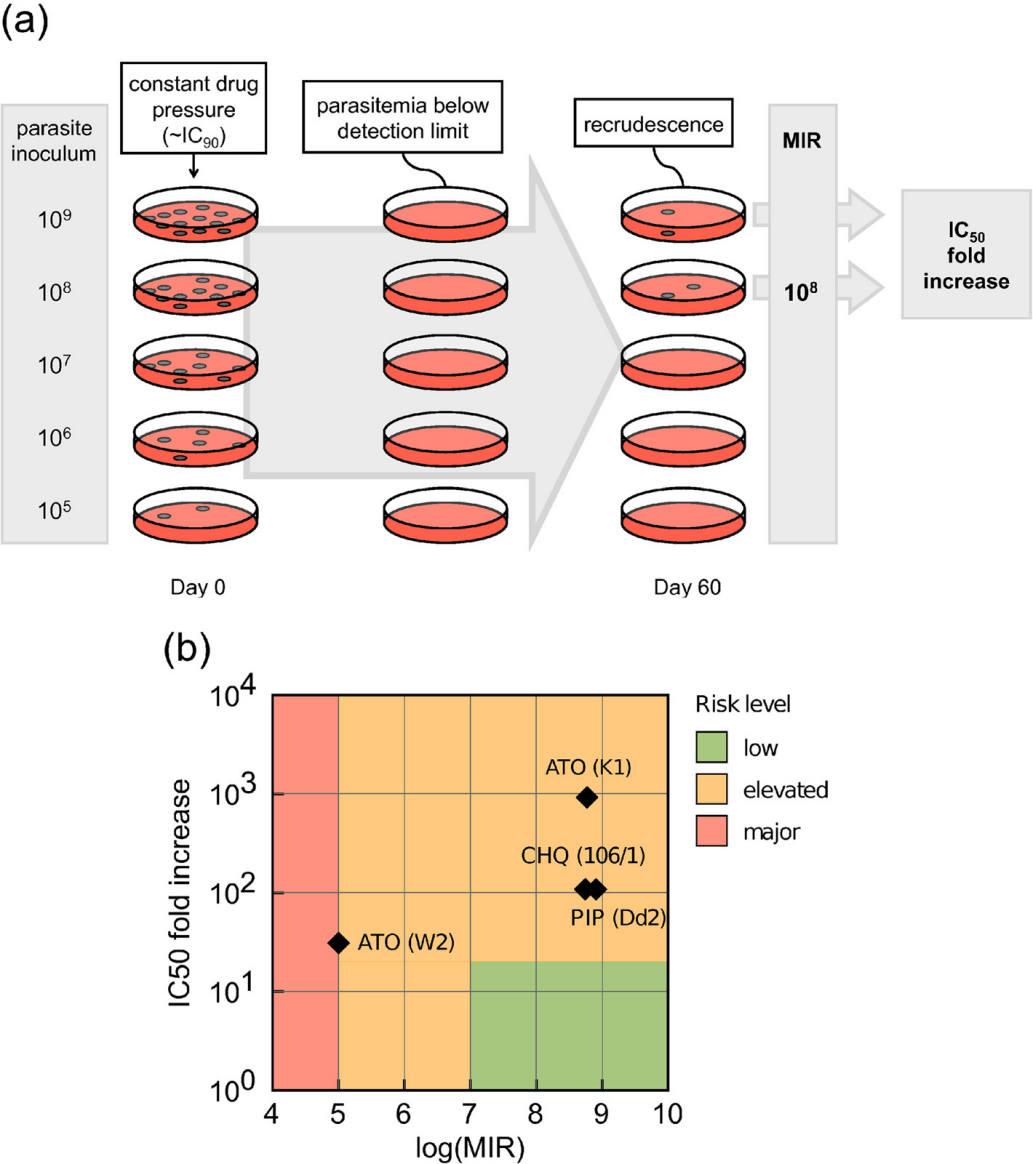
***In vitro *resistance selection assessment.** (**a**) A standard *in vitro* protocol for resistance selection frequency measurement uses defined starting inocula of a *P. falciparum* strain pressured with a constant level of drug nearing the IC_90_. Parasitemia falls below detection limits but eventual resistant parasites are able to recrudesce and to be cloned for subsequent determination of the IC_50_ fold increase. The minimal inoculum for resistance (MIR) is a measure of the resistance selection frequency, while the IC_50_ fold increase measures the level of resistance. (**b**) These two endpoints are used to classify anti-malarial compounds according to risk levels (see main text). It is advisable to run control experiments in parallel with compounds known to select resistance readily, such as atovaquone.

The simplest approach is *in vitro* selection of resistant parasites, which can be applied on a large number of compounds earlier in drug discovery. *P. falciparum* intraerythrocytic cultures, with starting inocula ranging from 10^5^ to 10^9^ parasites, are exposed to a concentration of the compound nearing IC_90_ and monitored during 60 days for recrudescent parasites (Figure 3a). The minimal inoculum for resistance (MIR) can be determined and is an indirect measure of the likelihood of a resistant genotype to occur and to be selected *in vitro*. In addition, the IC_50_ shift of the resistant mutants as compared to the parental sensitive strain is a measure of the resistance intensity [47-49]. Dd2 has an accelerated resistance to multiple drugs (ARMD) phenotype, possibly due to defective DNA repair mechanisms [47,50], and is therefore an ideal strain to investigate rare mutational events within the practical limits of *in vitro* culture volumes. Importantly, MIR from ARMD strains are assumed to be lower because of the general higher mutation rate of these parasites and not because they acquire alternative resistance mechanisms. Moreover, *ex vivo* results seem to be in good agreement with clinical experience. Atovaquone resistance is generated relatively easily in patients, and in laboratory experiments results either in low MIR or large IC_50_ increases [3,4,47]. Chloroquine and artemisinin derivatives, which escaped resistance clinically for a long time despite widespread use as monotherapy, do not readily produce stable resistant mutants in selection experiments [22]. 

A combination of MIR and IC_50_ increase in Dd2 can be used to flag compounds at high risk of resistance (Figure 3b). An MIR equal to or below 10^5^ is a major risk, as it suggests that only a single nucleotide mutation is sufficient. A MIR of 10^7^ would also be high risk, if combined with a greater than 20-fold shift in the IC_50._ Combinations of MIR and IC_50_ shift data available in the literature are presented in the Figure 3b and Table 3. This limited data set shows strain dependence of MIRs, atovaquone showing an MIR of 10^5^ for in W2 and at 6×10^8^ in K1 [4,47]. For example, the confirmation that cyclopropyl carboxamides had an MIR of less than 10^8^ in the standard 3D7 strain, and an IC_50_ shift of 100-fold range, gives two clear signals that the series should be put on hold, unless other redeeming features can be identified [51]. 

**Table 3:** MIR and associated IC50 fold increase reported in the literature

**Compound**	**Strain**	**Pressure**	**MIR**	**IC**_**50**_**fold increase**	**Mechanism**^**a**^	**Ref.**^**a**^
Atovaquone	W2	10×IC_50_	1×10^5^	30x	n/a	[47]
Atovaquone	K1	6×/16×IC_50_	6×10^8^	900x	single point mutations in *pfcytb*	[4]
Piperaquine	Dd2	2×IC_50_	8.5×10^8^	100x	63-kb fragment amplification	[49]
Chloroquine	106/1	3×IC_50_	6×10^8^	100x	single point mutations in *pfcrt*	[52]
GSK2645947	3D7	10×IC_50_	<10^8^	100x	n/a	[51]

^a^n/a:non available.

Mutations that give a selective advantage against drugs often cause a reduction in fitness in its absence [53,54]. A key fitness parameter is the relative asexual growth rate [55], measured *in vitro* by monitoring cultures seeded with the derived resistant and the original (sensitive) parental strain at a given ratio. The evolution of the strain ratio can then be monitored by DNA sequencing or qPCR [56-58]. The proportional loss of fitness (C) can be calculated from the change in strain ratio ΔR_n_, after *n* generations, where that value is equal to 1/(1-C)^*n*^[58]. If C ≥ 0 the sensitive strain will outgrow the resistant strain, however, if C<0 this would be major potential risk, suggesting that the strain may also out-compete the original wild type in humans. Of note, such a scenario has already been observed with *P. chabaudi* chloroquine and pyrimethamine resistant parasites displaying a fitness gain even in the absence of drug pressure [53]. A second key parameter of parasite fitness is its transmission potential, which depends not only on its growth rate but also on its ability to produce viable gametocytes. The effect of resistance can also be investigated on the sexual phase of the parasite cycle, using recently developed *in vitro* methods [59]. A resistant parasite unable to complete a sexual cycle is of no clinical relevance, as it cannot be transmitted.

*In vivo* resistance selection experiments allow a more physiological variation of the drug concentration and are more representative of the clinical situation [60]. For instance, the *pfcytb* Y268C mutation conferring atovaquone resistance in the wild could only be reproduced using a rodent *in vivo* model [61]. However, *in vivo* experiments do not allow to control the level of drug pressure or the number of parasite subjected to it and are not ideal to profile and cross-compare a large number of early compounds.

## Goal III: understanding the molecular mechanism of resistance

One key to understanding the importance of a resistant phenotype is its molecular characterization. Mutations in genes encoding pumps and transporters are concerning, since they could imply parallel resistance generation to a wide variety of different molecules. Point mutations may enable a link with the molecular target to be established. The genetic changes causing resistance can be identified using tiling arrays, full genome sequencing or linkage analysis and candidate genes can be validated using allelic exchange experiments [62-65]. The full genome sequences of a large number of *P. falciparum* parasites from diverse locations have already been determined [66]. For those molecules like NITD609 for which molecular markers of resistance have already been identified, it will also be important to examine these sequences and determine whether the target gene is highly polymorphic in these natural populations. This straightforward examination will give advance warning of any regions where resistant parasites might already by present, and easily selected by use of the drug.

Additionally, *in vitro* experiments performed to characterize the compound speed of action and its activity at various stages of the parasite lifecycle can be repeated using resistant parasites in order to better understand the resistance mode-of-action [59,67]. Recent advances in imaging also allow monitoring the intraerythrocitic life cycle of the parasites *in vitro* and *in vivo* and could similarly be used to compare resistant and sensitive parasites [68].

## Clinical and regulatory requirement

**Figure 4:**
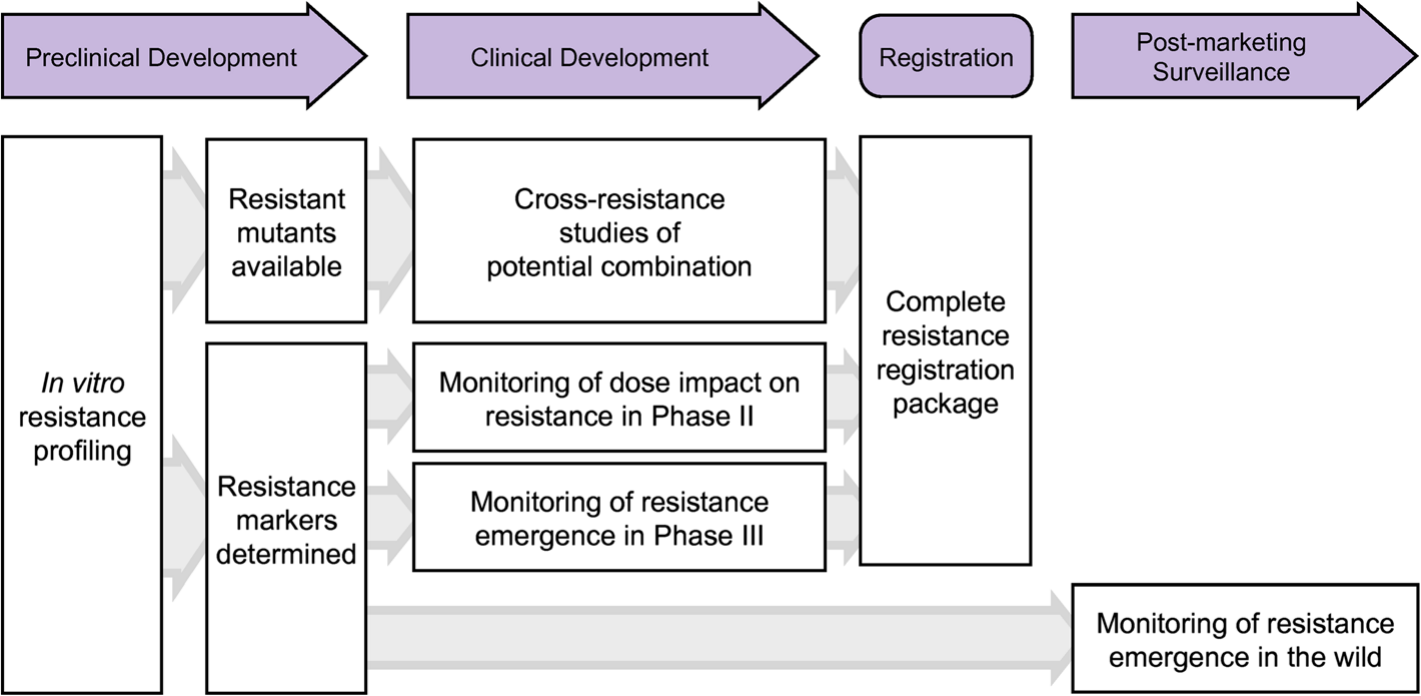
**Resistance profiling and clinical development.***In vitro* selection experiments typically generate resistant parasites from which resistance markers can be identified. This permits the identification of more robust combinations by assessing acquired and *de novo* cross-resistance studies with parasites already resistant to potential partner drugs. Resistance markers can be monitored during Phase II and III to include resistance selection as a clinical factor and to insure the appropriate resistance data package for registration. Post-marketing surveillance will also directly benefit from the a priori knowledge of resistance markers.

The development of technologies for assessing resistance means that comprehensive resistance testing should take place at all phases of drug development, including post-marketing pharmacovigilance (Figure 4). Ideally, the preclinical studies will have produced resistant strains and identified resistance markers, which are especially useful in the post-marketing surveillance phase. Resistance selection could also be performed for both partners of potential therapeutic combination using single-resistant mutants to evaluate the frequency of double-resistance, which should be equal or inferior to the single-resistance selection frequency. The identification of *in vitro* resistance markers allows careful monitoring for their potential appearance during clinical trials. Analysis of samples during dose ranging studies for resistance selection could have an impact on dose selection, but presumably only if the changes in the IC_50_ value were relatively minor. Systematic monitoring of resistance selection in Phase III will consolidate the data on frequency and help to consolidate the understanding of the development of resistance in different geographic and endemicity settings. This will be important information for the regulatory filings, for national malaria control programmes, and for coordinated efforts of resistance monitoring [69]. Regulators have universally recognized the importance of a systematic approach to the characterization of new compounds in regard to their resistance generation potential. The US-FDA recommend in their ‘Guidance for Industry for Malaria drug development’ that “the ability of *Plasmodium* strains to develop resistance when subjected to drug pressure should be examined in appropriate *in vitro* and/or *in vivo* models; this examination should include evaluating the potential for cross-resistance to drugs in the same class or in other classes. If resistance is demonstrated, it is important to identify the mechanism of resistance. Attempts should be made to evaluate the clinical significance of any changes in phenotype (e.g., *in vitro* susceptibility to the drug) or genotype observed in preclinical studies by correlating such changes with clinical outcome” [70]. 

Characterization of resistance potential has been mentioned in the registration summaries of all anti-malarials submitted stringent regulatory authorities in the past ten years: Coartem® (artemether-lumefantrine, Novartis) to the US-FDA; Eurartesim (dihydroartemisinin-piperaquine, Sigma-Tau) at the EMA; and more recently Pyramax (pyronaridine-artesunate, Shin Poong Pharmaceuticals) which obtained marketing authorization under the EMA’s article 58) [71-73]. The MMV strategy for resistance characterization will provide this information for the regulators and ensure a complete resistance registration package.

## Conclusion

The process presented here integrates information about the potential importance of resistance selection and spreading into the preclinical and clinical development programme for new anti-malarial drugs. At the preclinical level, the experimental framework requires a test cascade of six essential steps covered by three specific experimental procedures. The output of this workflow allows to classify the compound risk profiles as low, elevated or major (Figure 1 and 3b). This is one of the factors that will be discussed as part of the preclinical candidate selection process, a decision matrix which includes safety margin, and severity of safety signals observed, human predicted potency, speed of killing, effect on other lifecycle stages such as gametocytes and hypnozoites [74]. A major risk suggests that the compound will either face pre-existing resistance or will select new clinically significant mechanisms of resistance. This risk level should prevent further development of the compound, in the absence of a clear mitigating factor linked to major synergy in combination studies. An elevated risk is associated where there is a clear signal, and is an obvious concern. Provided that there are a small number of other elevated risks in the candidate selection matrix these compounds can be progressed, but resistance needs to be continually monitored in clinical studies, and will be a key factor in the selection of dose and combination partner. Risk mitigation is essential, making it important to understand the molecular mechanism, and to have genetic markers to survey its occurrence. A low risk classification suggests that resistance is unlikely to arise, although it is still important to continually monitor for changes in IC_50_ in the clinical program.

The outcome of resistance studies has several implications influencing decision making in drug development. First, the IC_50_ as part of the calculation for the prediction of human dose should include the range of potencies observed, and focus on the value from the most resistant parasites. This needs to be continually re-evaluated based on emerging data from primary field isolates. Second, potential cross-resistance amongst partners in drug combinations can be checked using laboratory derived resistant strains. Ideal drug combinations are these that regroup compounds driving opposed or even incompatible resistance mechanisms [75]. Third, the knowledge of *in vitro* identified mutations conferring resistance and susceptible genes allow monitoring during the large scale clinical trials in Phase III and post-launch, to give an early warning signal for the emergence of clinically significant effects.

The experimental strategy presented here also has limitations. It applies to compounds acting against *P. falciparum* asexual intraerythrocytic stage only. However, these represent the majority of the parasite biomass. Although the next generation of anti-malarials may include increased focus on the hepatic forms, understanding potential resistance selection at these lifecycle stages will be supported by knowledge of the resistance mechanisms in intraerythrocytic stages. Another limitation is the standardized approach itself. Resistance is a complex phenomenon that will require tailor-made studies. For instance, artemisinin resistance as currently observed does not translate into clear IC_50_ shift, rendering its study in cross-resistance and resistance selection experiments challenging [5]. However a standardized method does at least allow a common framework for comparing compounds and making development decisions.

The workflow presented here allows to measure the risks of resistance generation, the magnitude of the change in IC50, and the effect on transmission at an early stage in drug discovery. By determining the molecular mechanism of resistance, and identifying potential cross-resistance issues, high-risk chemical series can be de-prioritized, in a time and cost effective manner. The generation of a standard resistance profile of new drugs will supply common information to regulators and national control bodies to facilitate decision-making. This information will also allow for the focused monitoring of compounds after registration. This strategy will contribute to developing long-lasting anti-malarial drugs by identifying and monitoring robust compounds during their development.

## Competing interests

The authors declare that they have no competing interests.

## Authors’ contributions

Wrote the first draft of the manuscript and consulted with experts: XCD. Contributed to the writing of the manuscript: XCD, DU, TNCW. ICMJE criteria for authorship read and met: XCD, DU, TNCW. Agree with manuscript results and conclusions: XCD, DU, TNCW. All authors read and approved the final version of the paper.

## Author details

Medicines for Malaria Venture, 20 rte de Pré Bois, Geneva CH 1215, Switzerland.
